# Differentially expressed lnc‐NOS2P3‐miR‐939‐5p axis in chronic heart failure inhibits myocardial and endothelial cells apoptosis via iNOS/TNFα pathway

**DOI:** 10.1111/jcmm.15740

**Published:** 2020-08-25

**Authors:** Cuncun Chen, Ming Zong, Ying Lu, Yide Guo, Honggen Lv, Lihong Xie, Zhiyan Fu, Yu Cheng, Yuying Si, Bei Ye, Lieying Fan

**Affiliations:** ^1^ Department of Clinical Laboratory Shanghai East Hospital Tongji University School of Medicine Shanghai China

**Keywords:** apoptosis, chronic heart failure, INOS, LncRNA‐NOS2P3, microRNA‐939‐5p, TNFα

## Abstract

Inflammatory cytokine‐induced cell apoptosis is important for initiation and progression of chronic heart failure (CHF). Non‐coding RNAs, including long non‐coding RNAs and microRNAs, have emerged as critical regulators of this pathological process. The role in regulating inflammation and induction to cell apoptosis in CHF is not well understood. This study found CHF patients had elevated serum miR‐939‐5p, with greater increase in New York Heart Association (NYHA) I‐II patients than in NYHA III‐IV. Moreover, miR‐939‐5p was positively correlated with B‐type natriuretic peptide (BNP) in NYHA III‐IV patients, while not in NYHA I‐II. Further study showed miR‐939‐5p mimics promoted cell proliferation and inhibited inflammatory cytokine‐induced apoptosis of HUVECs and H9C2, while inhibition of endogenous miR‐939‐5p produced the opposite effects. Induced nitric oxide synthase (iNOS) and tumour necrosis factor α (TNFα) were identified as target genes of miR‐939‐5p. Additionally, lncRNA‐NOS2P3 acted as an endogenous sponge RNA to inhibit miR‐939‐5p expression, regulate the expression of iNOS/TNFα and control inflammation‐induced cells apoptosis. These suggest that CHF patients exhibited elevated serum miR‐939‐5p level especially in NYHA I‐II grades. And lnc‐NOS2P3‐miR‐939‐5p‐iNOS/TNFα pathway regulated inflammatory cytokine‐induced endothelial and myocardial cells apoptosis and provided a promising strategy for diagnosis and treatment of CHF.

## 1 INTRODUCTION

As more and more patients survive their initial cardiovascular diseases, chronic heart failure (CHF) becomes the leading cause of elderly hospitalization and death worldwide.^1^ Therapeutic strategies improvements didn’t increase CHF survival rate effectively.^2^ CHF is a progressive disease, and the critical pathogenesis is myocardial pathological remodelling, initially triggered by multiple signal and network interactions^3^ including overactivation of many neurohormones and inflammation factors after myocardial injury.^4^, ^5^ CHF is classified into four grades according to ESC guidelines, diagnosis and treatment in early grades would accept more ideal therapeutic effect.^6^

Circulating inflammatory cytokines predict clinical outcomes and dynamically changed during the process of CHF. Inflammatory cytokine‐induced myocardial and endothelial cells (ECs) apoptosis facilitates myocardial pathological remodeling.^5^ After heart injury, cardiomyocyte proliferation was stimulated to improve regenerative capacity.^7^ While the increasing rate of cardiomyocyte proliferation after injury remains insufficient to fully regenerate the lost myocardial tissue.^8^ Exception for myocytes, non‐myocytes of heart consists of >60% ECs,^9^ which presented a much higher frequency in direct implications on cardiac pathophysiology.^10^ Induced myocyte proliferation after MI would cause defective heart regeneration with newly formed myocyte unlikely to survive in the absence of ECs.^11^ Fast revascularization is essential to support cardiac regeneration.^12^ And the regeneration without vascularization ended prematurely in the injured area.^13^ To inhibit inflammation‐induced myocardial and ECs apoptosis in CHF is critical.

A large number of non‐coding RNAs will be activated during development of CHF, including microRNAs (miRNAs) and long non‐coding RNAs (lncRNAs).^14^ MiRNAs, 19‐25 nucleotides, modulate the physiological and pathological process in transcriptional or post‐transcriptional levels. MiRNAs induced by inflammation could regulate myocardial diseases. MiR‐155, containing in macrophage exosomes, suppressed fibroblast proliferation and promote fibroblast inflammation during cardiac injury.^15^ The miR‐146b‐TRAF6‐IL‐6/CCL2 (MCP‐1) axis promoted cardiac inflammation, fibrosis and ventricular dysfunction.^16^ Inhibition of miR‐103 attenuated inflammation and endoplasmic reticulum stress in atherosclerosis through disrupting the PTEN‐mediated MAPK signaling.^17^ But there are few studies between inflammation‐related miRNAs and heart failure. LncRNAs, exceeding 200 nucleotides, were reported to be closely related to CHF,^18^ and functioned as competing RNAs of miRNAs to regulate cardiac remodelling through transcriptional and post‐transcriptional levels.^19^, ^20^

Normal heart maintained low level of nitric oxide (NO) synthesized from Ca^2+^ dependent endothelial nitric oxide synthase (eNOS), inhibiting myocardial and ECs apoptosis to preserve cardiac normal physiology. However, inflammation activated Ca^2+^ independent inducible nitric oxide synthase (iNOS) would produce high level of NO, leading to myocardial and ECs apoptosis, and caused negative inotropic effect.^21^ The NO level in CHF patients was much higher.^22^ More, inflammatory TNFα was reported to promote iNOS production in mice.^23^ Further study found slightly increased level of iNOS and TNFα in NYHA I‐II grades CHF patients with no significance compared with normal controls, but was significantly up‐regulation in NYHA III‐IV patients.^24^ To specifically inhibit NO from iNOS and maintain its balance in CHF is essential. Guo et al identified iNOS as target gene of miR‐939‐5p in hepatocytes.^25^ And our microarray analysis also indicated iNOS may be its candidate target.^26^

Our previous study selected 11 miRNAs including miR‐939‐5p to predict the early virological response to interferon treatment in chronic hepatitis B (CHB) patients.^27^ The expression of miR‐939‐5p in hepatic perforation tissues of CHB patients was positively correlated with ALT level (*R* = .42, *P *< 0.05), and in immune activation group (ALT > 40 U/L) was higher than that in immune tolerance group (ALT < 40 U/L) (data not shown). Meanwhile, miRNA‐939‐5p restricted Hepatitis B virus by targeting Jumonji Domain‐containing 3 (JMJD3) mediated chromatin remodeling.^26^ Moreover, we found exsomes secreted from liver non‐parenchymal cells contained large amount of miR‐939‐5p when stimulated by IFN‐α.^28^ The activation of retinoic acid‐induced gene protein I (RIG‐I) pathway also produced a large amounts of miRNA‐939‐5p.^29^ Taken together, miR‐939‐5p was extensively related to chronic inflammation. However, there is no study between miR‐939‐5p and CHF.

This study we explored miR‐939‐5p expression in CHF patients and found serum miR‐939‐5p level in CHF patients elevated obviously compared to normal controls, with greater level in NYHA I‐II grades than NYHA III‐IV. And we investigated lnc‐NOS2P3‐miR‐939‐5p‐iNOS/TNFα pathway regulating inflammatory induced endothelial and myocardial cells apoptosis. Our study provided a promising strategy for early diagnosis and treatment of CHF.

## 2 MATERIALS AND METHODS

### 2.1 Study population and data collection

A total of 75 CHF and 36 non‐CHF control patients of Shanghai East hospital affiliated to Tongji University were collected from October 2017 to April 2018 for miR‐939‐5p level detection. And plasma of 40 CHF patients and 20 normal controls during Jun. 2020 was collected for detection of iNOS and TNFɑ. There were no significant varieties in sex composition and age. CHF was defined according to ESC Guidelines of heart failure in 2016^6^ as Class I patients, no symptoms and no limitation in ordinary physical activity, for example shortness of breath when walking and climbing stairs; class II, mild symptoms and slight limitation during ordinary activity; Class III, marked limitation in activity due to symptoms, even during less‐than‐ordinary activity and Class IV, severe limitations, experiences symptoms even while at rest. 41 NYHA I‐II patients and 24 NYHA III‐IV patients were included in this study. Major exclusion criteria were patients with renal failure (serum creatinine >176 μmol/L); patients given digitalis and diuretics within 24‐48 hours, and β‐blockers used in the same day.

Patients’ fasting venous blood samples were obtained for B‐type natriuretic peptide (BNP; analysed by Roche cobas e411) and the patient’s serum samples were stored at −80°C for RNA isolation and analysis of miR‐939‐5p expression by quantitative reverse transcriptase polymerase chain reaction (RT‐qPCR).

The Institutional Review Board of Tongji Medical School affiliated Shanghai East Hospital approved the study protocol and the written informed consent was obtained from each participant before any sample or data collection.

### 2.2 Cell culture

Human umbilical vein ECs (HUVECs, ATCC® CRL‐1730™) were grown in RPMI 1640 medium (GIBCO, Grand Island, NY, USA) supplemented with 10% foetal bovine serum (FBS) (GIBCO). H9C2 rat myocytes (ATCC, Rockvile, MD, USA) were cultured in Dulbecco’s modified Eagle’s medium (DMEM) (GIBCO) with 10% FBS.

### 2.3 RT‐qPCR and microarray analysis

Total RNA of serum samples and cultured cells was extracted by TRIzol (Life Technologies, Thermo Fisher Scientific, USA ). 600 μL TRIzol was added into 300 μL serum samples, and cel‐miR‐39 from RiboBio (Guangzhou, China) was added into the mixture as external reference. MicroRNAs were reverse transcribed using Bulge‐loop RT primer and subsequently quantified with miRNA qPCR primer sets (both from RiboBio). LncRNAs were reverse transcribed using random primer and oligo dT primer. One Step SYBR®_PrimeScript® PLUS RT‐RNA PCR Kit (TaKaRa, Biotechnology, Japan) was used for first‐strand cDNA synthesis and quantitative PCR analysis. MiRNAs expression levels were normalized to U6. The expression of lncRNAs and mRNAs was normalized to GAPDH.

To identify target transcript and interacted lncRNAs of miRNA‐939‐5p, cells were transfected with miR‐939 mimics (100 nM) for 36 hours and total RNA was harvested. CDNA microarray was performed with human LncRNA V2.0 from Arraystar Inc, which contains probes for protein‐coding transcripts and long non‐coding RNAs, following the recommended workflow. Differentially expressed genes with over twofold change were selected for further filtering and RT‐qPCR validation.

### 2.4 Target prediction analysis of the miRNA and lncRNA

Previous study suggested that iNOS was the candidate target gene of miR‐939‐5p according to microarray.^26^ And the bioinformatics RNAhybrid predicted iNOS as the target of miR‐939‐5p. TargetScan database predicts two conserved binding sites of miR‐939‐5p in TNFα 3′UTR.

Bioinformatics of DIANA‐lncBase, lncRNAMap, LncRNADisease and lncRNome database were used to predict lncRNAs targeting miR‐939‐5p combining the microarray results.

### 2.5 Western blotting

Total cell protein was extracted by RIPA lysis buffer (Beyotime, Shanghai, China), containing in protease inhibitors for 30 minutes at 4°C. The protein concentration was determined using Bradford Assay (Bio‐Rad, CA, USA). Total protein extracts of 20 μg were resolved by SDS‐PAGE gel electrophoresis and transferred to PVDF membranes (Bio‐Rad) for Western blot. Target protein was detected using specific primary antibody, and the primary antibodies were diluted as follows: Bcl2 (1:500, 2872S;1 Cell Signaling), Bax (1:500, 5023S, Cell Signaling), caspase3 (1:1000, SC7272, Santa Cruz), cyclinB1 (1:500, SC53236, Santa Cruz), iNOS (1:200, SC7271, Santa Cruz), β‐actin (1:2000, SC47778, Santa Cruz). Bound antibodies were detected by a peroxidase‐conjugated secondary antibody, and the signals were visualized using a chemiluminescence kit (Cell Signaling Technology). β‐actin protein levels were used as loading control.

### 2.6 Cell transfection and treatment

MiRNAs mimics, antagomirs and lncRNA smart silencers (RiboBio, Guangzhou, China) were transfected into cells using lipofectamine 3000 (Life Technologies) according to the manufacturer’s instructions. The medium was changed into 1%FBS RPI 1640/DMEM containing 2 μg/mL IL‐1β and 800 IU/mL IFN‐γ 12 hours later and cells would be collected for assays after treatment for 48 hours.

Lnc‐NOS2P3 expression plasmids and its mutants of miR‐939‐5p conserved binding sites were constructed into pIRES2‐EGFP plasmid (GENE, Shanghai, China), which were referred as lnc‐NOS2P3‐WT and lnc‐NOS2P3‐mut.

### 2.7 CCK8 cell viability assay

Cells were seeded in 48‐well culture plates and incubated at 37°C with 5%CO_2_. After treatment, the CCK8 assay (Dojindo, Japan) was performed. Briefly, 1/10 volume CCK8 solution was added to each well and incubated for 1‐2 hours. Then the supernatants were transferred to 96‐well plate and the optical density of each well was measured at 450 nm with a microplate reader (Bio‐Rad, CA, USA).

### 2.8 Apoptosis detection using Flow cytometry

HUVECs or H9C2 myocytes were washed in PBS and resuspended at the density of 5 × 10^5^ cells/mL with 500 μL binding buffer (1×). Afterwards, 5 μL Annexin V‐FITC solution and 5 μL propidium iodide (BD, NY, USA) were added into the suspension and the mixture was incubated for 15 minutes at room temperature avoiding light. Then, cells were performed with flow cytometry analysis.

### 2.9 Dual‐luciferase reporter assay

TNFα‐3′UTR (799 bp length) wide type containing miR‐939‐5p binding sites and its binding site mutation were cloned to pMIR‐REPORT Luciferase H306 reporter plasmids, named as TNFα 3′UTR‐WT/TNFα 3′UTR‐MUT (provided by ObiO, Shanghai, China). The whole length of lnc‐NOS2P3 and its mutant to miR‐939‐5p binding sites were constructed into GV272, named as lnc‐NOS2P3‐WT‐luc and lnc‐NOS2P3‐mut‐lnc (produced by GENECHEM, Shanghai, China).

HUVECs were seeded in 48‐well plate (3 × 10^4^ per well) and co‐transfected with 0.5 μg luciferase reporter plasmid, 0.01 μg Prl‐TK (expressing Renilla luciferase; Promega) and 100 nM miR‐939‐5p mimic or control. After 48 hours, cells were collected by passive lysis buffer and assayed for luciferase activity with Dual‐Luciferase Assay kit (Promega). Firefly luciferase activities were normalized to renilla luciferase activities.

### 2.10 Statistical analysis

All data were analysed using Graphpad Prism V5.0 and SPSS 18.0 statistical software and shown as standard error of the mean (SEM). Mann‐Whitney U test was used to analyse miR‐939‐5p level in clinical samples. For cell culture experiments, Student’s t test was used. The correlation analysis was performed by Spearman’s rank correlation. Data were considered significantly different if *P* < 0.05.

## 3 RESULTS

### 3.1 Serum level of miR‐939‐5p in various NYHA grades of CHF patients

**Figure 1:**
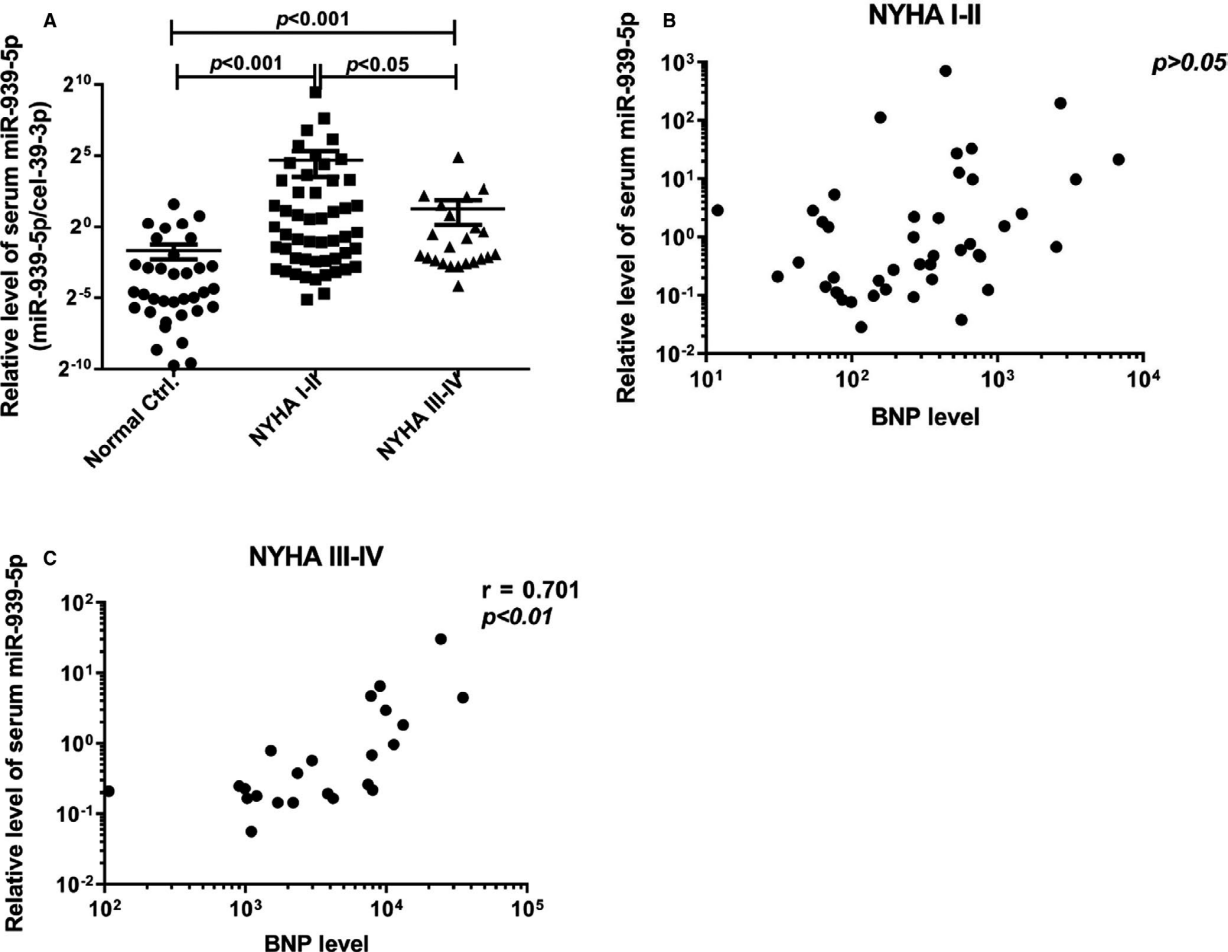
The serum expression of miR‐939‐5p was higher in chronic heart failure patients than normal people. A, The relative serum miR‐939‐5p expression was detected and compared in NYHA I‐II grade, NYHA III‐IV grade CHF patients and normal people by qRT‐PCR, adding cel‐39‐3p as external control. Data are mean ±SEM. ^*^ *P* < .05 and ^**^ *P* < .01. B and C, The correlation between BNP and miR‐939‐5p level in CHF patients were analysed by Spearman’s correlation. B, there is no obvious correlation in CHF NYHA I‐II patients (n = 41) *P *> 0.05. C, miR‐939‐5p expression in CHF NYHA III‐IV patients was significantly correlated with BNP level, (n = 24) *r* = .701, *P *< 0.01

To determine the role of miR‐939‐5p in CHF, we measured serum level of miR‐939‐5p in 75 CHF patients, with 51 NYHA I‐II and 24 NYHA III‐IV patients, compared with 36 non‐CHF control patients. The results showed that serum miR‐939‐5p level in CHF patients, including NYHA I‐II and NYHA III‐IV patients, was significantly higher than normal control. Moreover, the level in NYHA I‐II patients was obviously higher than in NYHA III‐IV (Figure 1A). Next, we analysed the correlation between miR‐939‐5p and plasma BNP, the commonly marker for CHF diagnosis. No significant correlation was observed in NYHA I‐II patients (Figure 1B). But miR‐939‐5p has high correlation with BNP in NYHA III‐IV, *r* = 0.701, *P *< 0.01 (Figure 1C). Usually, significant elevation of blood BNP suggests heart failure patients had entered into NYHA III or worse.^2^ Meanwhile, 100‐400 pg/mL BNP showed uncertainty in diagnosis of CHF, especially with no significant clinical symptoms.^30^ Serum miR‐939‐5p increased specifically in NYHA I‐II patients with no relation to BNP. It may predict the possibility for early diagnosis of CHF. Of course, it needs further verification.

Previous study has demonstrated that miR‐939‐5p was an inflammation‐related miRNA.^29^, ^31^ And in CHF progression, cytokines (such as TNFα, IL‐1β, IL‐6, galectin 3 and IFN‐γ) were obviously stimulated.^32^ Taken together, miR‐939‐5p could be induced by inflammatory cytokines during CHF progression, especially in early stage of CHF.

### 3.2 MiR‐939‐5p inhibited inflammation‐induced apoptosis of EC HUVECs and myocardial cell H9C2

Inflammatory cytokine‐induced cell apoptosis was the initial response to cardiac injury, which was important for cardiac remodelling contributing to CHF progression.^4^ IL‐1β and IFN‐γ have been shown to affect cardiac function by activating NO synthase activity in cardiac myocytes.^33^ Myocardial and ECs consisted of the majority of cardiac cells.^9^ Thus, in this study we investigate the regulation of miR‐939‐5p to inflammation‐induced apoptosis in endothelial and myocardial cells, HUVECs and H9C2. The cytokines mix (CM) was IL‐1β and IFN‐γ.

**Figure 2:**
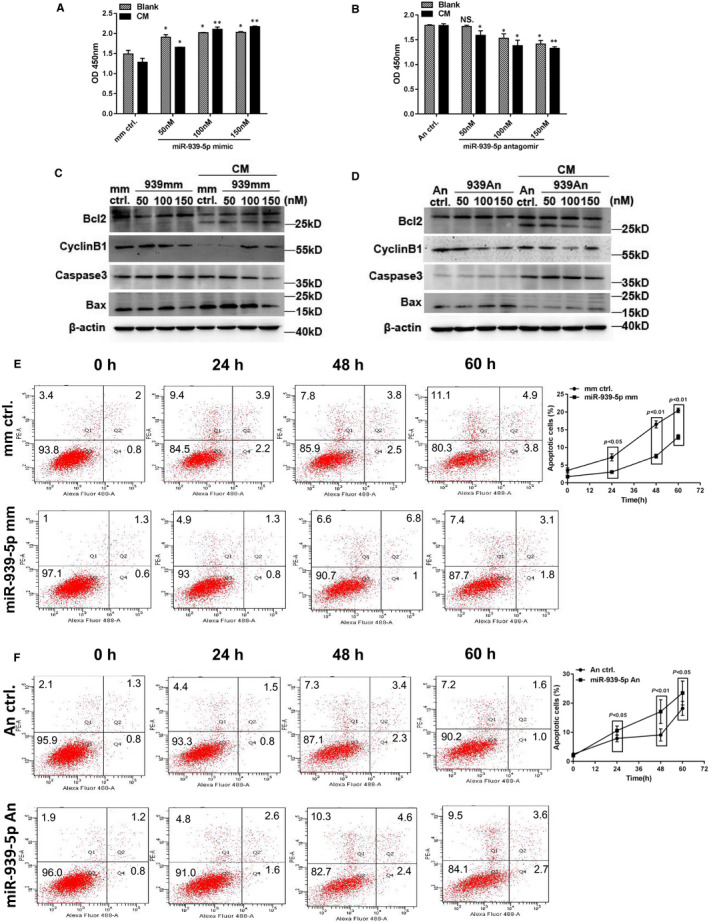
MiR‐939‐5p dose‐dependently regulated inflammation‐induced apoptosis in HUVECs. A‐D, miR‐939‐5p mimic or antagomir were transfected into HUVECs cells separately in 50, 100 and 150 nM, treated with or without CM of 2 ng/mL IL‐1β and 500 IU/mL IFNγ. A, Proliferation by CCK8 was dose‐dependently up‐regulated by miR‐939‐5p mimics in blank or CM treatment, mimic control as negative control. B, Proliferation was dose‐dependently down‐regulated by miR‐939‐5p antagomir compared to antagomir control. C, Apoptosis proteins by Western blot with miR‐939‐5p mimic and control. D, Apoptosis proteins by Western blot with miR‐939‐5p antagomir and control. E and F, Effect of miR‐939‐5p on apoptosis by flow chart at different time points, 0, 24, 48 and 60 h. E, miR‐939‐5p mimic vs. mimic control. F, miR‐939‐5p antagomir vs. antagomir control. Data are mean ± SEM. *P < 0.05 and **P < 0.01

CCK8 assay showed that HUVECs proliferation was dose‐dependently increased with transfection of miR‐939‐5p mimics from 50 to 150 nM (Figure 2A). In contrast, miR‐939‐5p antagomirs ranging from 50 to 150 nM significantly inhibited proliferation (Figure 2B). Further, miR‐939‐5p mimics elevated apoptosis inhibiting protein expression of Bcl2 and CyclinB1 in HUVECs under CM treatment. While the apoptosis promoting proteins Bax and caspase 3 decreased obviously (Figure 2C). On the contrary, miR‐939‐5p antagomirs resulted in down‐regulation of Bcl2 and CyclinB1 and up‐regulation of Bax and caspase 3 (Figure 2D). Moreover, flow cytometry by annexin V‐FITC/PI staining showed that miR‐939‐5p mimics inhibited CM induced HUVECs apoptosis in 0, 24, 48 and 60 hours with most significance in 48 hours (Figure 2E), and miR‐939‐5p antagomir facilitated the CM induced HUVECs apoptosis from 0 to 60 hours with most typically in 48 hours (Figure 2F), confirming the regulatory effects of miR‐939‐5p on inflammation‐induced cell apoptosis.

Similarly, we observed that miR‐939‐5p mimics promoted and antagomirs inhibited H9C2 myocytes proliferation by CCK8 assay (Figure S1A), and miR‐939‐5p regulated apoptosis of H9C2 cardiomyocytes the same as in HUVECs by Western blot (Figure S1B) and FACS analysis (Figure S1C).

Taken together, miR‐939‐5p could inhibit inflammation‐induced endothelial and myocardial cells apoptosis.

### 3.3 Identification of iNOS as a target of miR‐939‐5p

MiRNAs regulate gene expression to influence pathogenesis and biogenesis by inhibiting mRNA translation or promoting degradation.^34^ Previous study suggested that iNOS was a candidate target gene of miR‐939‐5p according to microarray and bioinformatics RNAhybrid analysis.^26^ INOS was predicted as miR‐939‐5p target gene in hepatocytes.^25^

**Figure 3:**
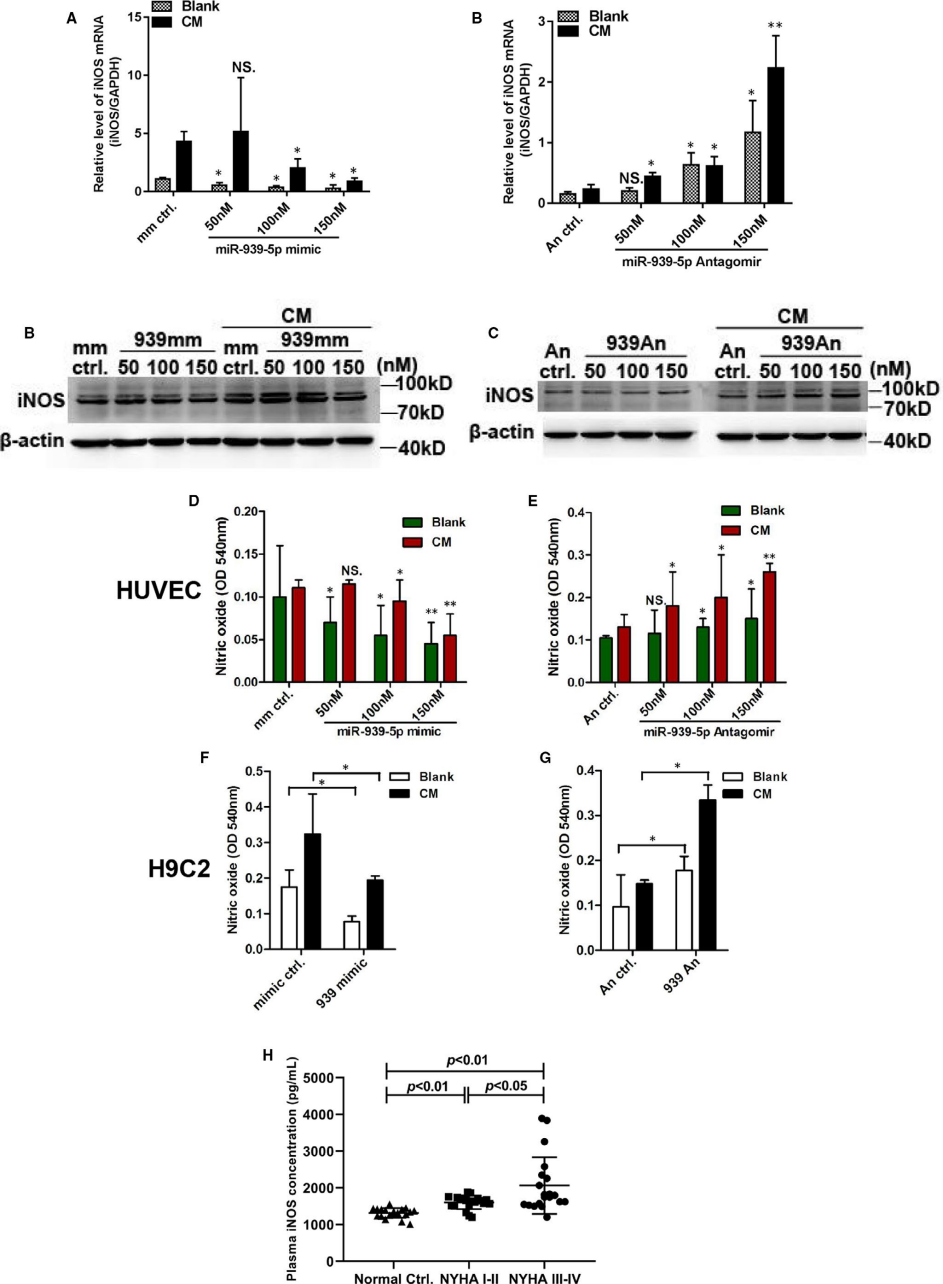
INOS was the validated target gene of miR‐939‐5p. A‐F, 50, 100 and 150nM miR‐939‐5p mimics or antagomirs were transfected into HUVECs with controls. The mRNA levels of iNOS by RT‐qPCR in blank or CM treatment, with miR‐939‐5p mimics and control, GAPDH as internal control (A), or with miR‐939‐5p antagomirs and control (B). C and D, Protein levels of iNOS with dose‐dependently expression of miR‐939‐5p mimic or antagomir and controls, β‐actin as internal control. C, miR‐939‐5p mimic dose‐dependently reduced protein level of iNOS. D, Knockdown of endogenous miR‐939‐5p dose‐dependently increased iNOS protein. E and F, The concentration of nitric oxide in culture supernatant was detected using Griess assay (OD 540 nm) (E), nitric oxide level elevated with dose‐dependent expression of miR‐939‐5p mimics vs. control. F, miR‐939‐5p antagomir reduced nitric oxide level. G, Nitric oxide level by Griess assay in H9C2 cells was inhibited by miR‐939‐5p. H, miR‐939‐5p antagomir raises the nitric oxide level in H9C2 cells. Data are mean ± SEM. *P < 0.05 and **P < 0.01. I, Plasma iNOS levels of CHF patients and normal controls were detected using ELISA. Data are mean ± SEM

INOS, inducible nitric oxide synthetase (also known as NOS2), activated dramatically by inflammation, induced massive NO synthesis which was critical in CHF progression.^35^ Whether iNOS could be regulated by miR‐939‐5p was confirmed. The results showed dose‐dependent down‐regulation of iNOS mRNA by miR‐939‐5p mimics in HUVECs (Figure 3A) and dose‐dependent up‐regulation of iNOS mRNA by miR‐939‐5p antagomirs (Figure 3B). Also, the protein level of iNOS expression was regulated by miR‐939‐5p mimics and antagomirs dose‐dependently according to Western blot (Figure 3C,D). Further, the nitrite oxide release by Griess assay showed that miR‐939‐5p mimic inhibited NO release into culture supernatants both in HUVECs and H9C2 (Figure 3E,G). While miR‐939‐5p anatogmirs promoted NO release in a dose‐dependent manner (Figure 3F,H). Meanwhile, the plasma iNOS levels in CHF NYHA I‐II increased slightly than normal controls by ELISA (Elabscience, Wuhan, China). And NYHA III‐IV patients were more higher than NYHA I‐II and normal controls (Figure 3I). These results demonstrated that miR‐939‐5p could regulate the target gene iNOS expression and the following NO synthesis.

### 3.4 Identification of TNFα as another target of miR‐939‐5p

Our microarray data showed reduced TNFα mRNA level in miR‐939‐5p mimic group, and TargetScan database predicts two conserved binding sites of miR‐939‐5p in TNFα 3′UTR. Meanwhile, TNFα could promote the expression of iNOS in mouse heart.^23^ Thus, we investigated whether TNFα was another target of miR‐939‐5p.

**Figure 4:**
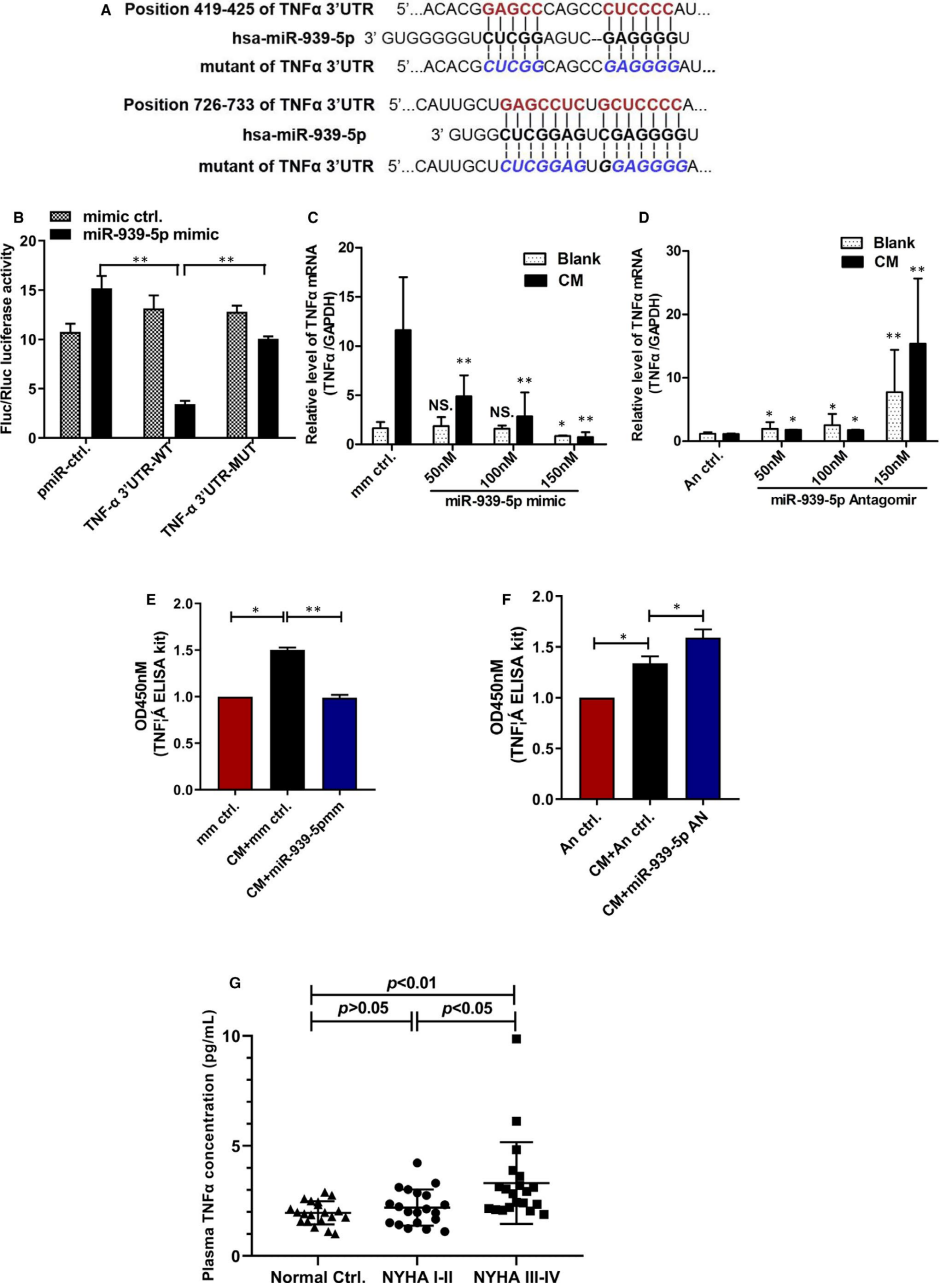
TNFα was another target gene of miR‐939‐5p. A, Putative miR‐939‐5p binding sites in the 3′UTR region of TNFα analysed by TargetScan program. Mutated miR‐939‐5p binding sites (TNFα‐3′UTR‐MUT) is shown. B, Luciferase constructs of TNFα‐3′UTR‐WT or TNFα‐3′UTR‐MUT were co‐transfected into HUVECs with miR‐939‐5p mimic or control. PGL3 served as a control. The luciferase activity was analysed. C and D, Different concentration from 50 to 150 nM of miR‐939‐5p mimics or antagomirs were transfected into HUVECs. C, miR‐939‐5p mimic reduced mRNA level of TNFα. D, miR‐939‐5p antagomir up‐regulated mRNA level of TNFα. E and F, TNFα in the cell culture supernatant was detected by ELISA. E, miR‐939‐5p mimic inhibited the level of TNFα vs mimic control. F, Knockdown of endogenous miR‐939‐5p promoted TNFα secretion. Data are mean ± SEM. *P < .05 and **P < .01. G, Plasma TNFα levels in CHF patients and normal controls were detected using CBA. Data are mean ± SEM

We cloned wide type 3′‐UTR sequence containing the two conserved binding sites (TNFα‐3′UTR‐WT) and mutant sequence with two mutated binding sites (TNFα‐3′UTR‐MUT) to generate luciferase reporter constructs (Figure4A). TNFα‐3′UTR‐WT or TNFα‐3′UTR‐MUT luciferase plasmids were co‐transfected with miR‐939‐5p mimics or mimics control into HUVECs. And results showed that miR‐939‐5p mimics reduced the luciferase activity of TNFα 3’UTR‐WT by about 75% versus mimic control, while no inhibitory effect was observed in mutant TNFα 3′UTR luciferase plasmid (Figure 4B). Then, increasing doses of miR‐939‐5p mimics or antagomirs were transfected into HUVECs. MiR‐939‐5p mimics inhibited TNFα mRNA in a dose‐dependent manner (Figure 4C). MiR‐939‐5p antagomirs led to a dose‐dependent increase of TNFα mRNA (Figure 4D). Moreover, ELISA assay confirmed the reduced TNFα level in cell supernatants by miR‐939‐5p mimics (Figure 4E) while the increasing TNFα secretion by antagomirs (Figure 4F). Moreover, the plasma TNFα levels in CHF NYHA I‐II were slightly higher than normal controls by Cytometric Bead Array (Celgene Biotechnology, Jiangxi, China). While NYHA III‐IV patients were obviously higher than normal controls and NYHA I‐II (Figure 4G). These results suggested that TNFα was another bonafide target of miR‐939‐5p.

### 3.5 Identification of candidate lncRNAs interacted with miR‐939‐5p

**Figure 5:**
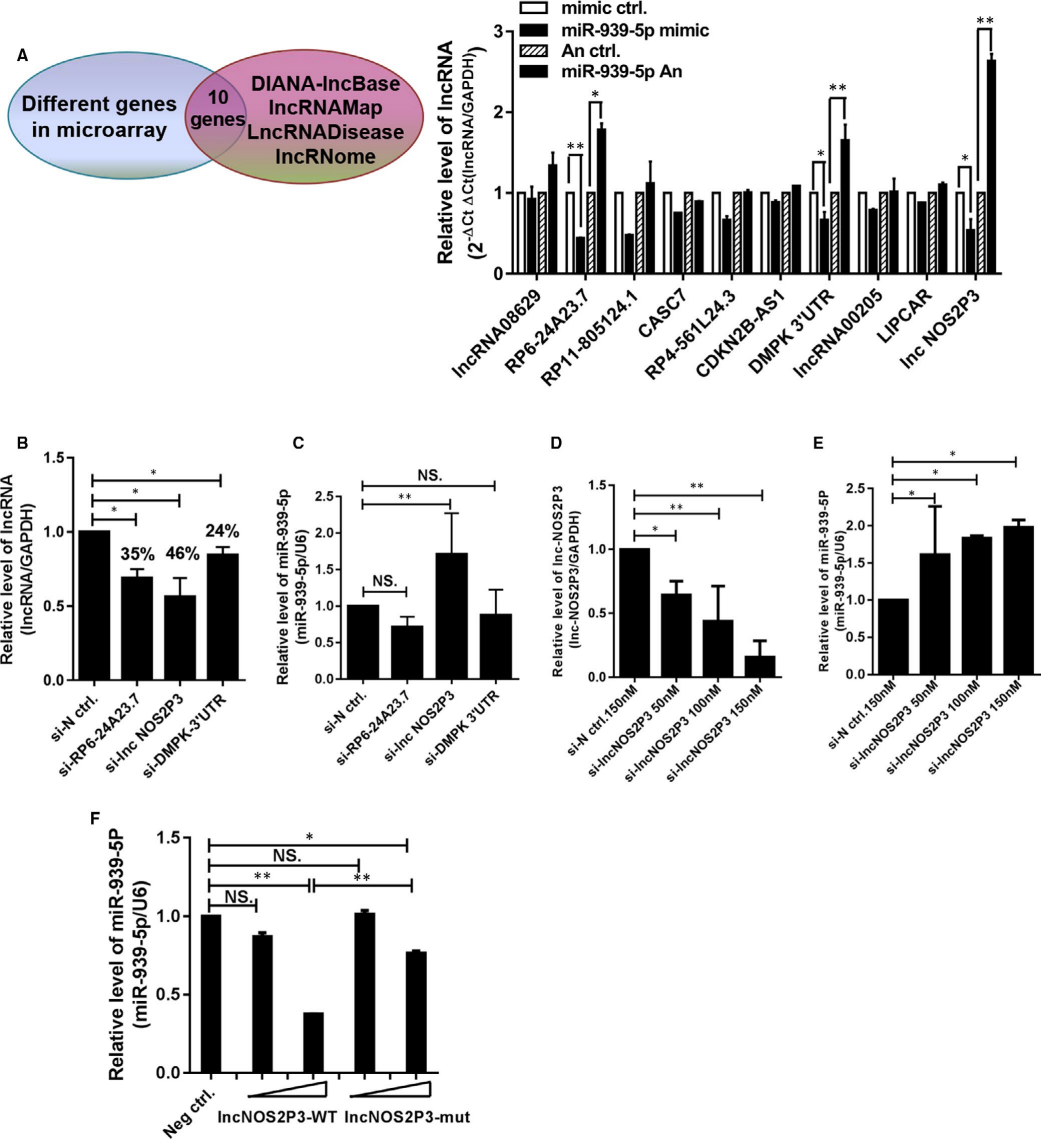
Selection of candidate lncRNAs interacted with miR‐939‐5p. A, 10 potential candidate lncRNAs from lncRNA‐databases filtration of differentially expressed genes in lncRNA microarray. 100 nM miR‐939‐5p mimic or antagomir and their controls were transfected into HUVECs. RNA was isolated and assayed by RT‐qPCR, results are presented relative to GAPDH (2^‐ΔΔCt^). B and C, 100 nM smart silencers of 3 lncRNAs (si‐lnc) were transfected into HUVECs. B, The interference effects were quantified by qRT‐PCR using lncRNAs specific primers from Ribobio, Guangzhou. C, MiR‐939‐5p was measured by qRT‐PCR, U6 as internal control. D and E, 50, 100, 150 nM smart silencers of lnc‐NO2P3 (si‐lnc‐NOS2P3) were transfected into HUVECs. D, The level of lnc‐NOS2P3 was determined. E, Relevant miR‐939‐5p levels were detected versus U6. F, 400 and 800 ng lnc‐NO2P3‐WT or lnc‐NO2P3‐mut and negative control were separately transfected into HUVECs, miR‐939‐5p levels were determined versus to U6. Data are mean ± SEM. *P < 0.05 and **P < 0.01

LncRNAs could act as miRNAs sponges to regulate their expression and target genes.^36^ Using microarray and bioinformatics by DIANA‐lncBase, lncRNAMap, LncRNADisease and lncRNome databases, we screened 10 lncRNAs as candidates regulating miR‐939‐5p (Figure 5A). By RT‐qPCR, 3 lncRNAs, RP6‐24A23.7, DMPK 3′UTR, NOS2P3, were selected to be reduced by miR‐939‐5p mimics and increased by antagomirs in HUVECs (Figure 5A), predicting possible interaction with miR‐939‐5p. For further confirmation, 3 lncRNAs were knocked down by smarter silencers. The knockdown efficiency was detected by RT‐qPCR (Figure 5B). Intriguingly, only the knockdown of lnc‐NOS2P3 led to the elevated miR‐939‐5p expression (Figure 5C). And when NOS2P3 level was dose‐dependently reduced by silencer‐lncNOS2P3 (Figure 5D), miR‐939‐5p level increased in a dose‐dependent manner (Figure 5E). To further verify the regulation of NOS2P3 to miR‐939‐5p, NOS2P3 wide type cDNA and mutant cDNA with two mutated conserved miR‐939‐5p binding sites were constructed into pIRES2‐GFP vectors (Figure S2A). The transfection of 800 ng lncNOS2P3‐WT significantly reduced miR‐939‐5p level, but with no same change by lncNOS2P3‐mut (Figure 5F). It suggested that lncRNA‐NOS2P3 might regulate miR‐939‐5p expression by direct base‐pair binding to miR‐939‐5p.

### 3.6 LncRNA‐NOS2P3 functioned as miR‐939‐5p sponge RNA and regulated the expression of target genes, iNOS and TNFα

NOS2P3, Nitric Oxide Synthase 2 Pseudogene 3, is the pseudogene 3 and the Ensembl number is ENSG00000230528 (from GeneCards). It localizes in chromosome17p11.2, the same chromosome with iNOS (NOS2). It is one of the three pseudogenes located in chromosome 17 neighbouring iNOS^37^ and its function has not been investigated yet.

**Figure 6:**
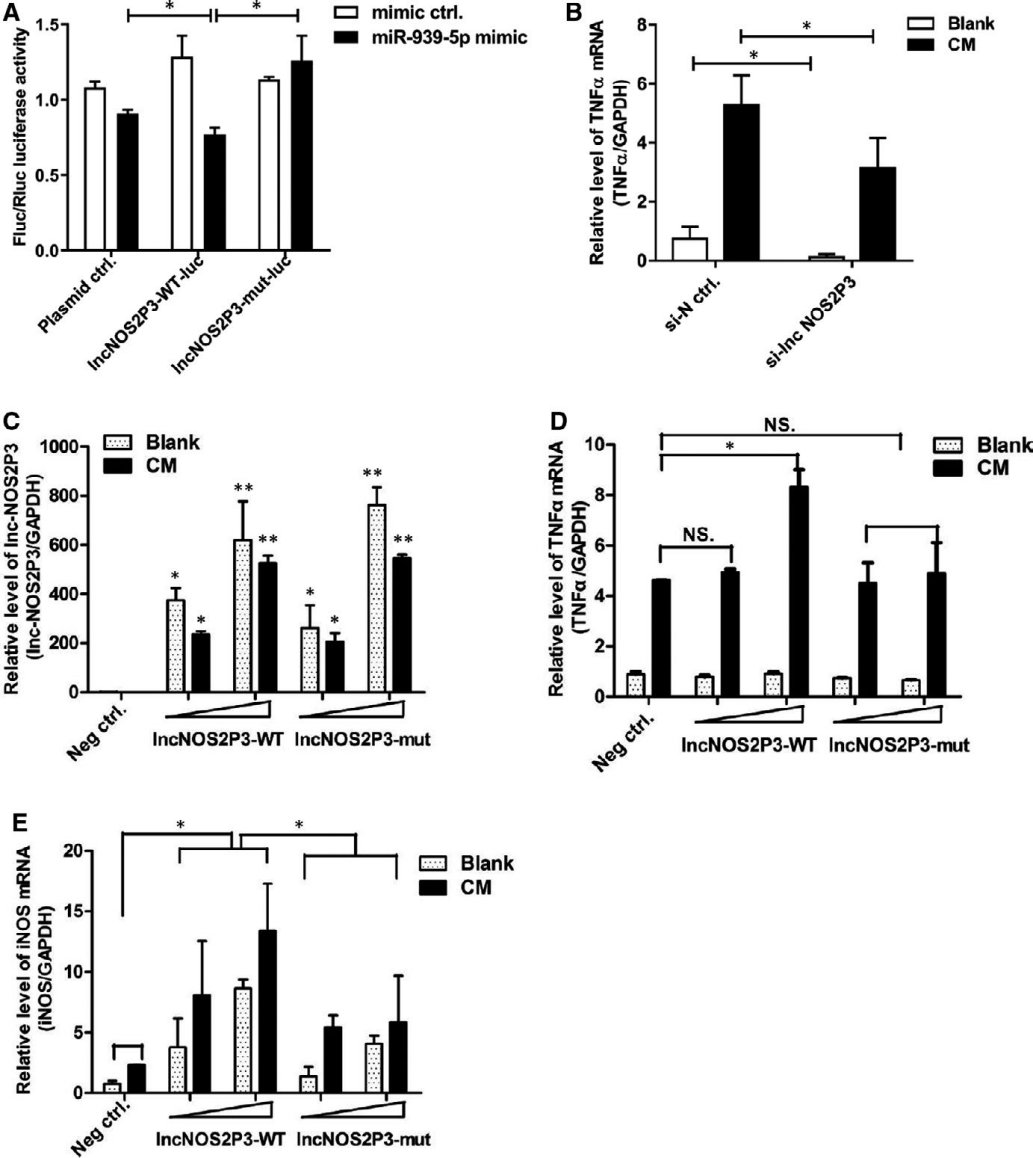
Lnc‐NOS2P3 was confirmed to regulate miR‐939‐5p and its target gene as sponge RNA. A, lnc‐NOS2P3 wide type and its mutant luciferase plasmids were co‐transfected into HUVECs with 100nM miR‐939‐5p mimic and control. Relative luciferase was obtained. B, TNFα mRNA was obviously reduced after transfection of 100nM smart silencer of lnc‐NOS2P3. C‐E, 400 and 800 ng lnc‐NOS2P3‐WT or lnc‐NOS2P3‐mut GFP plasmid and their negative control were transfected into 24 plates with or without CM. C, The increasing expression of lnc‐NOS2P3 and its mutant were detected by lnc‐NOS2P3 by RT‐qPCR. GAPDH acted as control. D, Relative TNFα mRNA level was measured 48 hours after treated with or without CM. E, Relative iNOS mRNA level was obtained 48 hours later. Data are mean ± SEM. *P < 0.05 and **P < 0.01

To verify direct binding of NOS2P3 to miR‐939‐5p, NOS2P3 cDNA was cloned into the downstream of luciferase construct Rluc‐GV272, with mutant cDNA construct of two conserved binding sites generated (Figure S2B). MiR‐939‐5p mimics significantly decreased lnc‐NOS2P3‐WT luciferase activity, but had no similar influence to lnc‐NOS2P3‐mut (Figure 6A).

With knockdown of endogenous NOS2P3 by si‐lnc NOS2P3, TNFα mRNA level induced by CM obviously decreased (Figure 6B). Next, we overexpressed lncNOS2P3‐WT and lncNOS2P3‐mut dose‐dependently in HUVECs (Figure 6C) and found TNFα mRNA increased dose‐dependently treating with increasing dose of lncNOS2P3‐WT, but had no effect by lncNOS2P3‐mut (Figure 6D). Similarly, iNOS mRNA level increased dose‐dependently following treatment with increasing dose of lncNOS2P3‐WT, but not by lncNOS2P3‐mut (Figure 6E). It suggests that NOS2P3 and miR‐939‐5p have base‐pairing interaction, which could influence the expression of target genes iNOS and TNFα.

### 3.7 Lnc‐NOS2P3 regulated inflammatory cytokine‐induced apoptosis through miR‐939‐5p

Although NOS2P3 interacts with miR‐939‐5p and regulates its expression and target genes, whether NOS2P3 affected inflammation‐induced apoptosis regulated by miR‐939‐5p was unknown.

**Figure 7:**
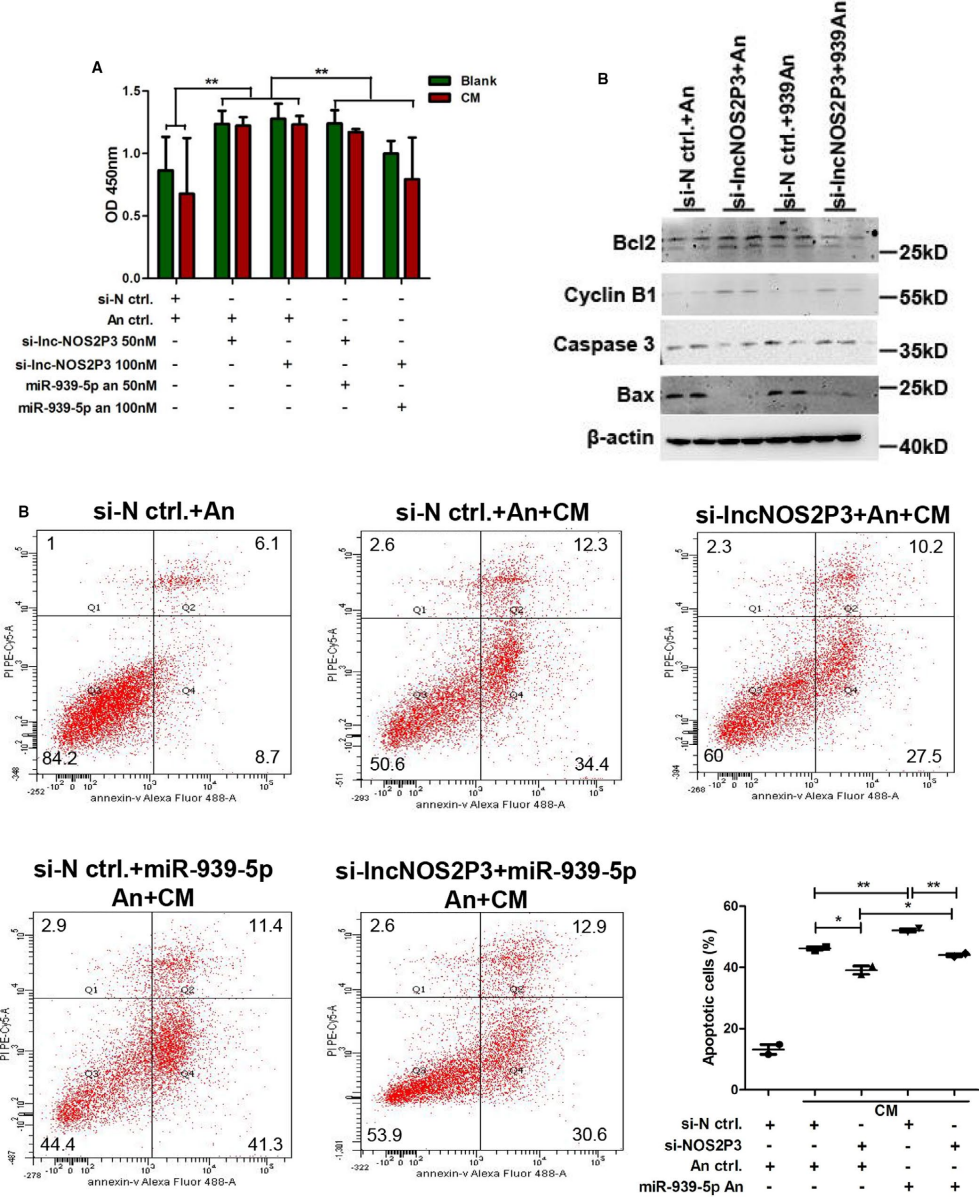
Knockdown of endogenous lnc‐NO2P3 inhibited the inflammation‐induced apoptosis which could be rescued by miR‐939‐5p antagomir. A, 50 or 100 nM si‐lnc‐NOS2P3, 50 or 100 nM miR‐939‐5p antagomir (miR‐939‐5p an), si‐negative control (si‐N ctrl.), and antagomir control (An ctrl.) were transfected into HUVECs as groups shown. Cell proliferation was got by CCK8 assay. B, Apoptosis cells were detected by flow cytometry in three replicate samples. The experiment was repeated twice and data were analysed statistically. C, Apoptosis related proteins were detected by Western blot in various groups. Data are mean ± SEM. *P < 0.05 and **P < 0.01

NOS2P3 was knocked down or overexpressed and then rescued with miR‐939‐5p antagomirs or mimics. Silencer‐lnc‐NOS2P3 increased the proliferation of HUVECs, but miR‐939‐5p antagomirs reversed it (Figure 7A). Further flow cytometry analysis showed that the knockdown of endogenous NOS2P3 significantly reduced the CM induced apoptosis, but miR‐939‐5p antagomirs rescued the reduction of apoptosis (Figure 7B). Moreover, Western blot confirmed that silencer‐lnc‐NOS2P3 up‐regulated the apoptosis inhibiting proteins Bcl2 and Cyclin B1 and inhibited the apoptosis promoting proteins Caspase 3 and Bax, and miR‐939‐5p antagomir reversed the expression of these proteins (Figure 7C).

**Figure 8:**
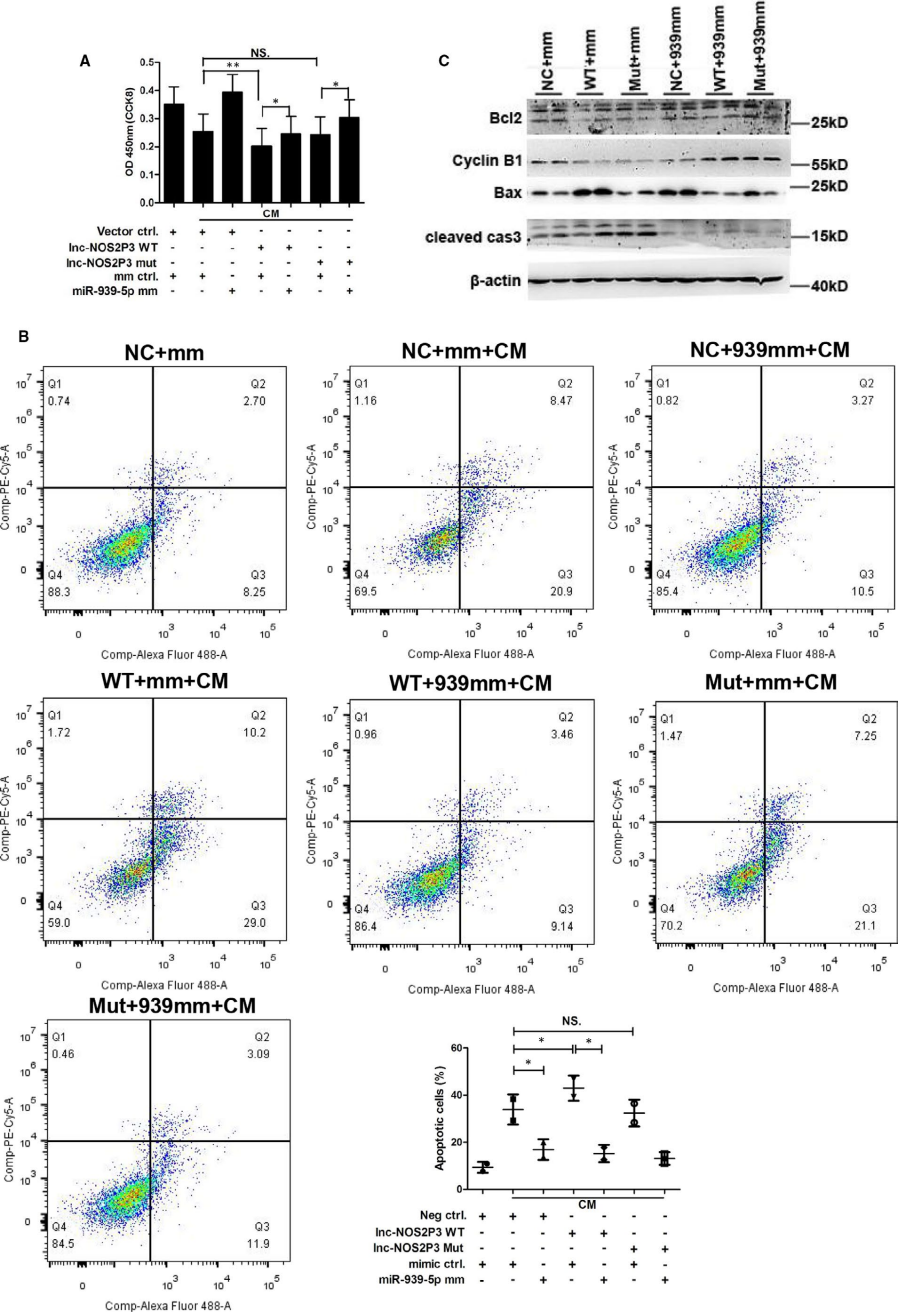
Overexpression of lnc‐NOS2P3 promoted inflammation‐induced apoptosis which was rescued by miR‐939‐5p mimic. A, 800 ng lnc‐NOS2P3‐WT or lnc‐NOS2P3‐mut and vector control were transfected into HUVECs with 100 nM miR‐939‐5p or mimic control. CCK8 assay showed its proliferation 48 hours later. B and C, 800 ng lnc‐NOS2P3‐WT (WT) or lnc‐NOS2P3‐mut (Mut) and their vector control (NC) were transfected with 100 nM miR‐939‐5p mimic (939 mm) or its mimic control (mm). B, Flow cytometry showed the apoptosis cells ratios, and data were analysed statistically. C, Apoptosis related proteins were measured by Western blot. Data are mean ± SEM. *P < 0.05 and **P < 0.01

Next, for further confirmation, we transfected lnc‐NOS2P3‐WT and lnc‐NOS2P3‐mut with miR‐939‐5p mimics. Lnc‐NOS2P3‐WT significantly reduced the proliferation of HUVECs, while miR‐939‐5p mimics rescued it. However, lnc‐NOS2P3‐mutant didn’t change the proliferation of HUVECs (Figure 8A). Flow cytometry displayed that lnc‐NOS2P3‐WT (WT) aggravated CM induced apoptosis compared to negative control plasmid (NC), which was reversed by miR‐939‐5p mimics. No significant change was observed by lnc‐NOS2P3‐mutant (Mut) (Figure 8B). Western blot further verified that lnc‐NOS2P3‐WT decreased the expression of apoptosis inhibiting proteins and increased apoptosis promoting proteins expression, which were reversed by miR‐939‐5p mimics, but the mutant had no obvious affection (Figure 8C). Collectively, NOS2P3 could regulate the cell apoptosis induced by inflammatory cytokines through miR‐939‐5p.

## 4 DISCUSSION

Early pathogenesis of CHF is abnormal gene expression induced by neurohormones and inflammation, followed with myocardial and ECs apoptosis.^38^ The attempt to prohibit early inflammation after heart injury is critical for early diagnosis and treatment. Here, we explored abnormal miR‐939‐5p expression in various CHF stages and analysed its relation with BNP. And the mechanism was that miR‐939‐5p inhibited cytokine‐induced myocardial and ECs apoptosis according to target genes iNOS and TNFα. Furthermore, lncRNA NOS2P3 was explored to regulate miR‐939‐5p expression as a sponge RNA and influenced miR‐939‐5p and target genes expression, controlling cytokine‐induced cells apoptosis.

We found that serum miR‐939‐5p level elevated in CHF patients than normal controls, greater in NYHA I‐II patients than in NYHA III‐IV. Further, in NYHA III‐IV patients, significant correlation between miR‐939‐5p and BNP was found, while not in NYHA I‐II. NYHA I‐II patients normally have mild or no clinical symptoms and are always neglected.^2^ Although BNP is the most common diagnostic marker, once massive plasma BNP was detected, patients always have entered into NYHA III grade or worse.^2^ BNP performs well to roll out, but less well to role in. Moreover, it’s unclear to select optimal cut‐points of BNP with the influence factors like age and renal functions.^39^ Serum miR‐939‐5p especially increased in NYHA I‐II patients and was not related to BNP, indicating the possibility in early CHF diagnosis. In future, it needs further verification on randomized controlled trial with more patients.

Previous research showed a robust induction of miR‐939‐5p by LPS or cytokine mix stimulation in primary human hepatocytes.^25^ And miR‐939‐5p may regulate pro‐inflammatory genes.^40^ Heart failure correlated extensively with serum pro‐inflammatory cytokines.^41^, ^42^ Yet the causative role of inflammation in disease progression is not well defined. The initial insult of CHF is mediated by inflammatory cytokine‐induced apoptosis^5^ and the interaction between apoptotic cardiomyocytes and microvascular network of ECs post‐MI has been studied extensively.^23^ Our study showed that miR‐939‐5p could influence cytokine‐induced myocardial and ECs apoptosis. Inhibition of ECs apoptosis may protect heart microvasculars. And the reduction of myocardial cell apoptosis could prevent the process of CHF.

Target genes iNOS and TNFα are key factors in NO system and CHF process. When initial insult occurred, inflammatory/cytokines of heart would induce miR‐939‐5p expression, controlling low level of iNOS and TNFα in NYHA I‐II stages which may induce myocardial and ECs apoptosis.^21^ As CHF processing, more and more inflammatory cytokines erupted, miR‐939‐5p was down‐regulated and was not enough to inhibit iNOS and TNFα. Massive iNOS activation and high concentration NO production would induce abundant myocardial and ECs apoptosis, heart will enter into decompensation stage of NYHA III‐IV with typical clinical symptoms.

Relative balance between pathological inflammatory pathways and tissue reparative processes (physiological inflammation) defined the trajectory of HF development.^43^ Single application of immunosuppressant didn’t achieve the desired therapeutic effect on CHF.^5^ Although elevated TNFα level leads to worsening HF,^32^ the net result of TNFα blockade using soluble receptor infusion (etanercept) or humanized neutralizing antibodies (infliximab) was negative.^44^, ^45^ It is worth exploring new therapy focusing on modestly lowering TNFα levels. And lncRNA‐miRNA network may be a promising choice. MiR‐939‐5p‐iNOS/TNFα axis provided a new strategy in reversing the pathological inflammatory for treatment of CHF.

LncRNAs participated in a wide range of diseases, such as nervous system diseases, cancer and cardiovascular diseases,^46^, ^47^, ^48^ also could function as ceRNA of the miRNAs influencing heart failure.^49^ LncRNA‐ROR promoted cardiac hypertrophy through miR‐133.^50^ Down‐regulated lncRNA‐GASL1 in CHF regulated cardiomyocyte apoptosis.^51^ Interfering of lncRNA ANRIL reduces heart failure in rats with diabetes by inhibiting myocardial oxidative stress.^52^ Lnc‐NOS2P3, the pseudogene 3, localizes in the same chromosome 17 neighbouring iNOS (NOS2). Its function has not been reported now. We selected that lnc‐NOS2P3 could regulate miR‐939‐5p expression according to act as a sponge RNA. Further, NOS2P3 influenced miR‐939‐5p target gene iNOS and TNFα, affecting inflammation‐induced apoptosis of myocardial and ECs. The spatial adjacency of NOS2P3 and iNOS indeed provided the possibility of functional interaction with each other, and possibility to miR‐939‐5p‐iNOS/TNFα pathway.

There existed the possibility that in early stage of CHF, NOS2P3 was lowly expressed to release miR‐939‐5p and inhibited target genes iNOS and TNFα and following NO synthesis, which prohibited myocardial and ECs apoptosis. To late stage, increasing expression of NOS2P3 inhibited miR‐939‐5p level and resulted in extensive NO synthesis and cell apoptosis. Moreover, the expression of lnc‐NOS2P3‐mut did not completely reverse the regulation to miR‐939‐5p compared to lncNOS2P3‐WT. Cause, in addition to acting as ceRNA, lncRNAs have been reported to modulate process of diseases through interacting with DNA or proteins.^34^ Maybe other regulation pathways existed.

Although tens of thousands of non‐coding RNAs have been found in humans, lots of lncRNAs and miRNAs do not show high interspecies conservation.^53^ Although research showed that LPS and CM injection could induce miR‐939‐5p expression, the iNOS expression could not be inhibited in mouse or rat.^25^ And we failed to find a homologous sequence of NOS2P3 and miR‐939‐5p in mice and rats in databases. Thus, functions of miR‐939‐5p and lnc‐NOS2P3 were not verified in vivo yet. Many studies have proved other factors of lncRNA conservation, such as structure, function and expression syntenic loci.^54^, ^55^, ^56^ Future in vivo validation may be performed in advanced evolved mammalian.

In conclusion, this study found the elevation of miR‐939‐5p in CHF, greater in NYHA I‐II patients than NYHA III‐IV. And the regulation of lnc‐NOS2P3‐miR‐939‐5p to iNOS/TNFα pathway and NO synthesis could restrict inflammation‐induced myocardial and ECs apoptosis, which may prohibit the process of CHF and provide a novel strategy for treatment of CHF.

## CONFLICT OF INTEREST

The authors confirm that there are no conflicts of interest.

## AUTHOR CONTRIBUTIONS

**Cuncun Chen:** Conceptualization (lead); Funding acquisition (lead); Project administration (lead); Writing‐original draft (lead); Writing‐review & editing (lead). **Ming Zong:** Conceptualization (equal); Data curation (supporting); Formal analysis (supporting). **Ying Lu:** Resources (supporting); Software (supporting); Supervision (supporting). **Yide Guo:** Investigation (supporting); Methodology (supporting); Project administration (supporting). **Honggen Lv:** Resources (supporting); Validation (supporting); Visualization (supporting). **Lihong Xie:** Formal analysis (supporting); Funding acquisition (supporting); Validation (supporting). **Zhiyan Fu:** Formal analysis (supporting); Resources (supporting); Software (supporting). **Yu Cheng:** Data curation (supporting); Resources (supporting); Visualization (supporting). **Yuying Si:** Conceptualization (supporting); Project administration (supporting); Validation (supporting). **Bei Ye:** Methodology (supporting); Project administration (supporting); Validation (supporting). **Lieying Fan:** Project administration (equal); Resources (equal); Supervision (equal); Visualization (equal).

## Supporting information

Fig S1‐S2

## Data Availability

I confirm that my article contains a Data Availability Statement even if no data are available (list of sample statements) unless my article type does not require one.
