# Supplementary material for: Differentially expressed lnc‐NOS2P3‐miR‐939‐5p axis in chronic heart failure inhibits myocardial and endothelial cells apoptosis via iNOS/TNFα pathway
Source: J Cell Mol Med. 2020 Aug 25;24(19):11381–96. doi: 10.1111/jcmm.15740 (PMC7576245; doi:10.1111/jcmm.15740)

Supplementary figure 1

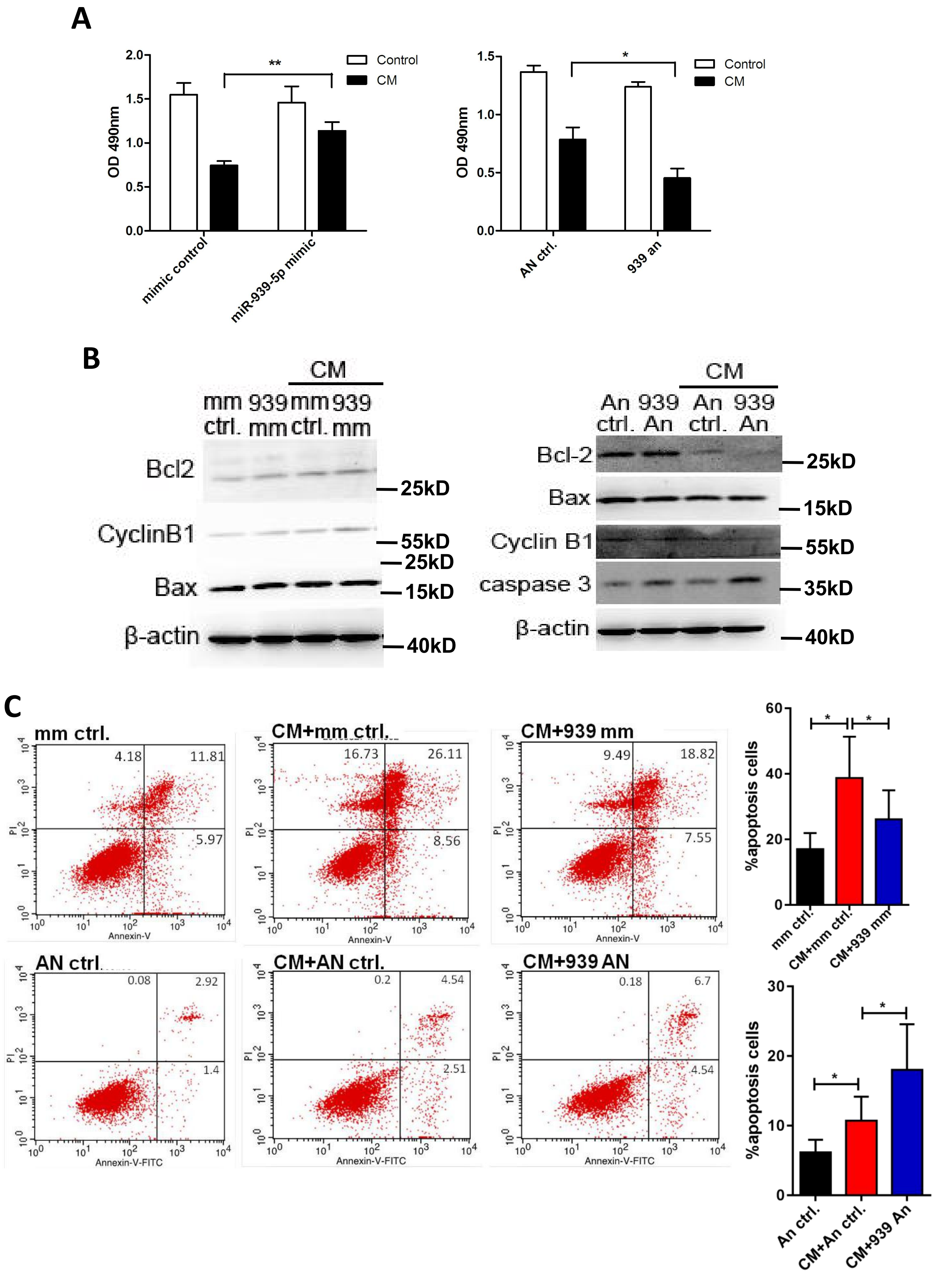

Supplementary figure 2

A

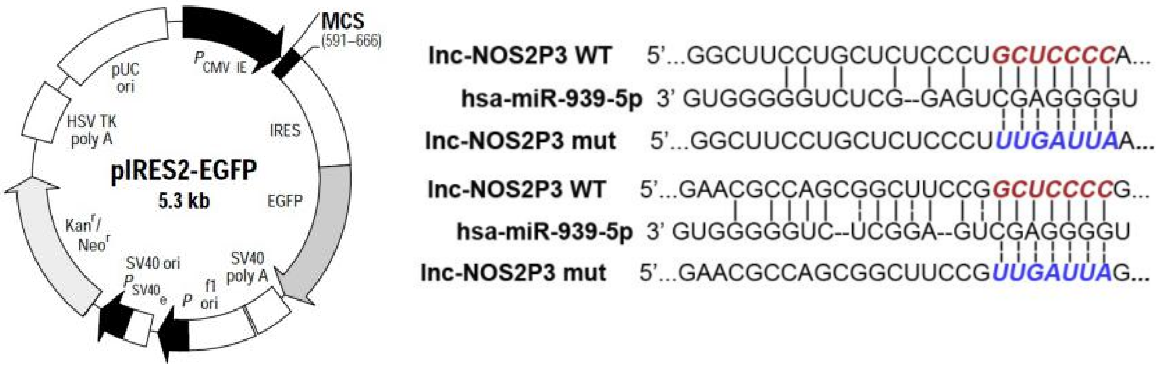

B

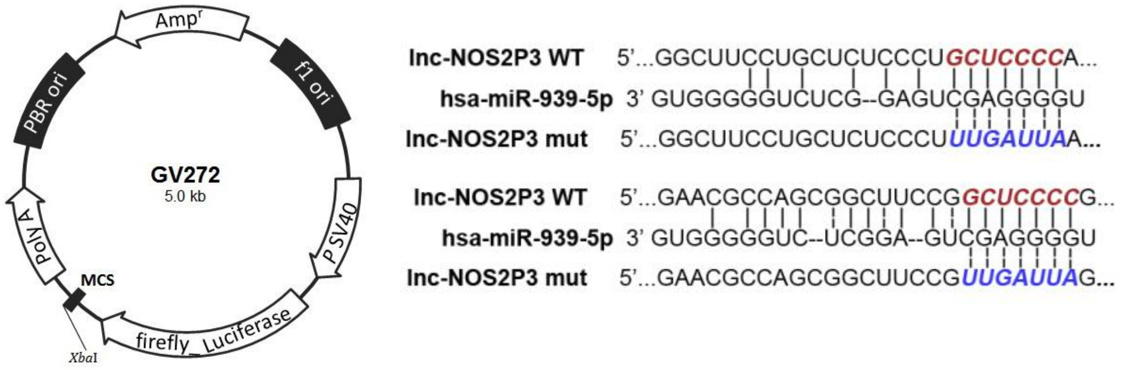

Supplement: Supplementary file 1 — Fig S1‐S2 [file JCMM-24-11381-s001.pdf]
